# Myeloid‐Derived Growth Factor Improves Endothelial Progenitor Cell Function and Angiogenesis in Mice With Diabetic Hindlimb Ischemia by Activating Akt/HIF‐1α Signalling Pathway

**DOI:** 10.1111/jcmm.71287

**Published:** 2026-07-14

**Authors:** Wen Mei, Biying Meng, Bilin Zhang, Yi Shu, Haizhao Luo, Jingwen Ye, Yijiang Hou, Biao Zhu, Jiajia Zhang

**Affiliations:** ^1^ Department of Endocrinology, School of Medicine the Sixth Affiliated Hospital of South China University of Technology (Nanhai District People's Hospital of Foshan) Foshan China; ^2^ Department of Endocrinology General Hospital of Central Theater Command Wuhan China; ^3^ Department of Stomatology, Fuxing Hospital Capital Medical University Beijing China

**Keywords:** angiogenesis, diabetic limb ischemia, endothelial progenitor cells, myeloid‐derived growth factor

## Abstract

Myeloid‐derived growth factor (MYDGF) offers new insights into inflammation and endothelial function, yet its role in endothelial progenitor cells (EPCs) and diabetes‐related hindlimb ischemia (DLI) remains unclear. In this study, we found that MYDGF depletion impaired the angiogenic function of EPCs, leading to reduced vascularisation and decreased blood flow in diabetic mice with hindlimb ischemia. Conversely, treatment with adeno‐associated virus–mediated MYDGF restored vascularisation and improved blood perfusion. In vitro, treatment with recombinant MYDGF attenuated EPC dysfunction, apoptosis and inflammatory responses. Mechanistic studies revealed that these protective effects were mediated via the AKT/HIF‐1α pathway, and silencing HIF‐1α abrogated the benefits of MYDGF both in vivo and in vitro. Together, these findings identified MYDGF as a potential therapeutic target for DLI.

## 1 Introduction

Diabetes mellitus (DM) is a chronic, complex metabolic disorder resulting from impaired insulin secretion or insulin resistance [1]. Prolonged hyperglycemia in DM leads to multiple clinical complications associated with metabolic syndrome [2]. Among these, DLI represents a severe manifestation of diabetic macrovascular disease. Patients with DLI typically exhibit more advanced arterial disease and poorer clinical outcomes compared with non‐diabetic individuals, often resulting in substantial physical disability, reduced productivity and significant emotional burden [3]. The pathogenesis of DLI is largely driven by impaired angiogenic responses to ischemia. Current therapeutic strategies, including revascularisation procedures and pharmacological interventions, frequently fail to achieve satisfactory outcomes due to the compromised angiogenic capacity in DM [4]. Therefore, there is an urgent need for novel therapies that can enhance angiogenesis and restore tissue perfusion in ischemic limbs under diabetic conditions.

Angiogenesis, the formation of new capillaries from pre‐existing blood vessels, is a fundamental physiological process that is crucial for restoring blood flow following injury [5]. This process is primarily driven by ischemia and ischemia‐induced transcription factors, such as hypoxia‐inducible factor‐1α (HIF‐1α), as well as downstream target genes, including vascular endothelial growth factor (VEGF) [6]. Therapeutic angiogenesis has been explored through cytokine administration to stimulate vessel formation or via transplantation of progenitor cells, such as bone marrow–derived endothelial progenitor cells (BM‐EPCs), which differentiate into endothelial cells (ECs) and secrete proangiogenic factors [7]. Both vascular ECs and EPCs are essential contributors to vascular repair and angiogenesis, yet they are highly susceptible to hyperglycemia‐induced damage [8, 9]. Among the various mechanisms underlying endothelial dysfunction, inflammation is recognised as a central contributor and a potential therapeutic target in cardiovascular diseases and DM [10]. Restoring the function of ECs and EPCs is therefore critical to counteract hyperglycemia‐induced impairment of angiogenesis in diabetic patients.

MYDGF is a novel secreted protein with potent anti‐apoptotic and tissue‐repairing properties. It has been implicated in a range of conditions, including cardiovascular disease, metabolic disorders, inflammatory diseases, kidney injury and cancer, where it exerts diverse biological effects [11]. Our previous work demonstrated that MYDGF inhibited inflammation, alleviated endothelial injury and mitigated atherosclerosis [12]. Moreover, MYDGF modulated neutrophil interstitial motility and inflammation via the hypoxia‐inducible factor 1 alpha (HIF‐1α) pathway in response to tissue damage [13]. HIF‐1α, in turn, regulates downstream proteins involved in glucose metabolism and angiogenesis, such as VEGF and erythropoietin, to facilitate adaptation to ischemia [14]. The ability of MYDGF to regulate EC function suggests its potential therapeutic value for DLI and impaired angiogenesis. In this study, we investigated whether MYDGF could restore ischemic angiogenesis under diabetic conditions and explored the underlying mechanisms.

## 2 Materials and Methods

### 2.1 Animals and Treatments

Animal care and experimental procedures were conducted in accordance with a protocol approved by the Animal Ethics Committee of the General Hospital of Central Theatre Command (Wuhan, China). MYDGF knockout (KO) and wild‐type (WT) littermate mice were generated by mating MYDGF^+/−^ breeders on a C57BL/6J background, purchased from Shanghai Model Organisms Centre Inc. (Shanghai, China) [12, 15]. To induce DM, mice were fed a high‐fat diet (HFD; Research Diets, D12492, USA) and subsequently administered streptozotocin (STZ; Sigma‐Aldrich, St Louis, MO, USA) according to established protocols [16]. Briefly, the WT and KO mice were both fed an HFD for 16 weeks. At 14 weeks of dietary regimen, mice were intraperitoneally injected with STZ (50 mg/kg per day) for 5 consecutive days to generate a mouse model of type 2 diabetes. Blood glucose levels were measured from tail‐vein samples, and only mice with glucose levels > 16.6 mmol/L were considered diabetic and included in experiments. To evaluate the effects of MYDGF in the diabetic model, WT and KO diabetic mice were randomly assigned to four groups: WT‐GFP, WT‐MYDGF, KO‐GFP, KO‐MYDGF (*n* = 8 per group). For adeno‐associated‐virus (AAV) treatment, MYDGF cDNA (GenBank accession number NM_080837.2) was synthesised and adeno‐associated virus (AAV)‐MYDGF was constructed as previously described [15]. Mice received a single tail‐vein injection of AAV‐MYDGF or control AAV‐green fluorescent protein (GFP) at a dose of 1 × 10^12^ viral genomes. Plasma MYDGF level was measured in duplicate by targeted LC–MS assay as previously described [15, 17]. To explore the potential mechanism of MYDGF, KO‐MYDGF diabetic mice were further randomised to two groups: KO‐MYDGF+ Ad‐siRNA Control (siCon) and KO‐MYDGF + Ad‐siRNA HIF1α (siHIF‐1α) (*n* = 8 per group). We synthesised HIF‐1α‐specific siRNA (siHIF‐1α; GenBank accession number NM_024359) (Table S1) and a scrambled control siRNA (siCon) oligonucleotides and cloned them into pSuper vector as previously described [18]. For Ad‐siHIF‐1α treatment in vivo, mice received a single injection of Ad‐siHIF‐1α at a dose of 1 × 10^10^ PFU through the tail vein.

### 2.2 Hindlimb Ischemia Surgery and Assessments

Hindlimb ischemia was induced in mice using optimised surgical procedures [18]. Blood flow was assessed with laser Doppler perfusion imaging (LDPI) (Perimed, Stockholm, Sweden) immediately after femoral artery excision and on postoperative days 7 and 14. Perfusion was quantified by calculating the ratio of blood flow in the ligated limb to that in the non‐ligated limb to reduce variability. Ambulatory impairment was evaluated using the following scoring system: 0, plantar and toe flexion present in response to gentle tail traction; 1, plantar flexion without toe flexion; 2, absence of plantar or toe flexion; 3, complete lack of foot use.

### 2.3 Capillary and Arteriole Density in Diabetic Ischemic Hindlimbs

Mice were euthanised 14 days after treatment, and adductor muscle samples were collected for histological analysis. Capillary and arteriole density were assessed by immunostaining tissue sections with CD31 (Servicebio, Wuhan, China) and α‐smooth muscle actin (α‐SMA; Boster Bio‐Engineering), following previously published protocols [16]. Vessel densities were quantified using ImageJ software.

### 2.4 Determination of Circulating EPCs and Homing Population of EPCs in Mice

The number of circulating EPCs was quantified by flow cytometry as previously described, with minor modifications [18]. Briefly, total mononuclear cells were isolated from peripheral blood using density gradient centrifugation with Histopaque‐1083 (Sigma‐Aldrich, St. Louis, MO, USA) and incubated with fluorescein isothiocyanate–labelled CD34 (FITC‐CD34; Invitrogen) and phycoerythrin‐labelled kinase insert domain receptor (PE‐KDR; Invitrogen) antibodies. To assess EPC homing, adductor muscle sections from ischemic limbs were immunostained with CD34 and KDR (R&D Systems) for fluorescence analysis.

### 2.5 BM‐EPCs and In Vitro Cell Culture

BM‐EPCs were isolated, cultured and characterised following previously established protocols [19]. EPCs for in vitro experiments were obtained from non‐diabetic WT mice (6–8 weeks old) and cultured as described in our prior reports [18].

### 2.6 Apoptosis Assay

Apoptosis was measured by flow cytometry (Beckman‐Coulter, Indianapolis, IN, USA) using annexin V‐FITC and propidium iodide (PI) staining (eBioscience, San Diego, CA, USA) according to the manufacturer's instructions. Apoptotic cells were defined as Annexin V^+^/PI^+^ (quadrant 2) and Annexin V^+^/PI^−^ (quadrant 3).

### 2.7 In Vitro Trans‐Endothelial Migration (TEM) Assay and Angiogenesis Assay

TEM, a critical function of EPCs in angiogenesis, was evaluated using a transwell assay. EPCs were cultured for 48 h at 37°C, and the number of cells migrating from the upper to lower chamber was quantified by independent investigators blinded to treatment. In vitro angiogenic capacity was assessed using a Matrigel tube formation assay, in which EPCs were incubated at 37°C with 5% CO_2_ for 12 h to form capillary‐like structures, following established protocols [20].

### 2.8 Inflammatory Analysis In Vivo and In Vitro

For in vivo inflammation assessment, 16 gastrocnemius muscle sections (4 per group) were collected on postoperative day 14. EPCs were divided into the normal glucose (NG, 5.5 mmol/L) group, high glucose (HG, 25 mmol/L) group, HG + recombinant MYDGF (rMYDGF, 100 ng/mL) group and HG + rMYDGF + small interfering RNA targeting HIF‐1α (siHIF‐1α) group. EPCs were pretreated with or without rMYDGF for 24 h and then incubated with or without HG for an additional 24 h. For siHIF‐1α experiments, EPCs were pretreated for 24 h before rMYDGF treatment. Lysates from gastrocnemius muscle tissue and supernatants from EPC cultures were collected and stored at −80°C. Protein levels of TNF‐α, IL‐6, and IL‐1β were measured using ELISA kits according to the manufacturer's instructions (R&D Systems, Minneapolis, MN, USA).

### 2.9 Western Blot Assay

Western blotting was performed as previously described [12]. Primary antibodies included protein kinase B (AKT), phospho‐AKT (p‐AKT), AMP‐activated protein kinase α (AMPKα), phospho‐AMPKα (p‐AMPKα), extracellular signal‐regulated kinases 1/2 (ERK1/2), phospho‐ERK1/2 (p‐ERK1/2), HIF‐1α, VEGF, stromal cell‐derived factor‐1α (SDF‐1α), Bax, Bcl‐2, cleaved caspase‐3 (all from Cell Signalling Technologies, USA) and β‐actin (Boster Bioengineering, China).

### 2.10 Statistical Analysis

Data were presented as mean ± standard error of the mean (SEM). Statistical analyses were conducted using SPSS 27.0 (IBM Corp., Armonk, NY, USA). Comparisons between two or multiple groups were performed using one‐way analysis of variance (ANOVA) with least significant difference (LSD) post hoc tests when equal variances were assumed or Dunnett's T3 post hoc tests when variances were unequal. Homogeneity of variance was assessed using Levene's test. A two‐sided *p* value < 0.05 was considered statistically significant.

## 3 Results

### 3.1 MYDGF Was Suppressed Under Diabetic Conditions, Whereas MYDGF Activation Facilitated Blood Flow Recovery in DLI

MYDGF has been shown to promote angiogenesis and wound healing in murine hearts following acute myocardial infarction [21]. Moreover, our previous studies demonstrated that serum MYDGF levels were reduced in both diabetic patients and diabetic mice [15]. However, the role of MYDGF in post‐ischemic blood flow recovery under diabetic conditions remains unclear. We therefore examined whether MYDGF expression is altered in diabetes and whether it contributes to vascular recovery following ischemic injury. A diabetic mouse model was established using HFD feeding followed by STZ administration, and DLI was induced by ligation of the left femoral artery (Figure S1A). Serum MYDGF protein levels were subsequently assessed. Consistent with our previous observations, serum MYDGF levels were significantly reduced in diabetic mice compared with non‐diabetic controls (Figure S1B). LDPI confirmed successful induction of ischemia and revealed that diabetes markedly impaired post‐ischemic blood flow recovery in the affected hindlimbs (Figure S1C,D). These findings led us to hypothesise that MYDGF plays a critical role in regulating perfusion recovery and tissue repair following DLI.

**FIGURE 1:**
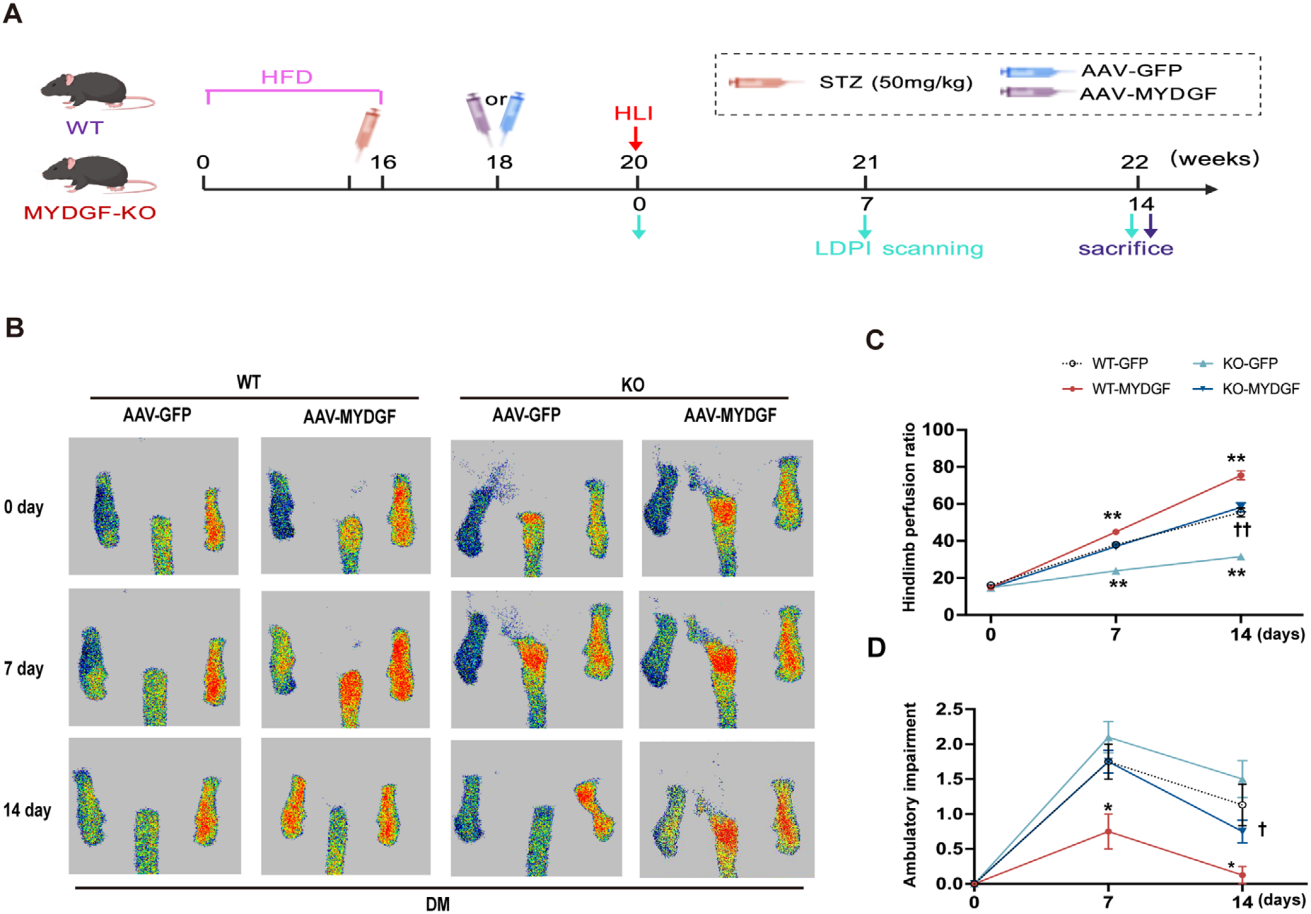
MYDGF facilitates blood flow recovery in diabetic mice subjected to hindlimb ischemia. (A) Experimental schematic illustrating the establishment of diabetic mice, administration of AAV and the procedure for femoral artery ligation. (B) Representative images illustrating blood flow in the hindlimb paws at 0, 7 and 14 days post‐ligation (*n* = 8 mice). (C) Quantification of blood flow recovery at 0, 7, and14 days post‐ligation, as assessed by the ischemic to non‐ischemic limb perfusion ratio. (D) Ambulatory impairment was scored at each time point. Values are presented as mean ± SEM in (C) and (D). *p < 0.05, **p < 0.01 versus WT‐GFP group; ^†^ *p* < 0.05, ^††^ *p* < 0.01 versus KO‐GFP group.

To directly investigate whether MYDGF could alleviate DLI, HFD/STZ‐induced DLI models were generated in WT‐MYDGF and KO‐MYDGF mice. Mice were randomly assigned in a 1:1 ratio to receive either AAV‐GFP (WT‐GFP and KO‐GFP) or AAV‐MYDGF (WT‐MYDGF and KO‐MYDGF) (Figure 1A). Serum MYDGF levels confirmed the absence of MYDGF expression in KO mice and demonstrated significantly elevated MYDGF levels in AAV‐MYDGF–treated mice compared with AAV‐GFP controls (Figure S1E).

Two weeks after viral administration, femoral artery ligation was performed, followed by serial assessment of perfusion recovery and molecular analyses at the indicated time points (Figure 1A). MYDGF deficiency markedly exacerbated ischemic injury and impaired blood flow recovery compared with diabetic WT mice. In contrast, blood flow recovery in the ischemic hindlimb was significantly enhanced in WT‐MYDGF and KO‐MYDGF mice relative to their respective AAV‐GFP controls (Figure 1B,C). Functional recovery was further evaluated using an ambulatory impairment scoring system. MYDGF‐deficient mice showed more severe limb dysfunction than WT mice at 14 days post‐ligation. Notably, both WT‐MYDGF and KO‐MYDGF mice displayed significantly improved limb function compared with WT‐GFP and KO‐GFP controls (Figure 1D). Collectively, these results indicate that MYDGF is suppressed under diabetic conditions and that restoration of MYDGF activity markedly improves blood perfusion and functional recovery in DLI, supporting its potential as a therapeutic target.

### 3.2 MYDGF Restoration Enhanced Vascularisation and Promoted EPC Mobilisation and Homing in Diabetic Ischemic Hindlimbs

**FIGURE 2:**
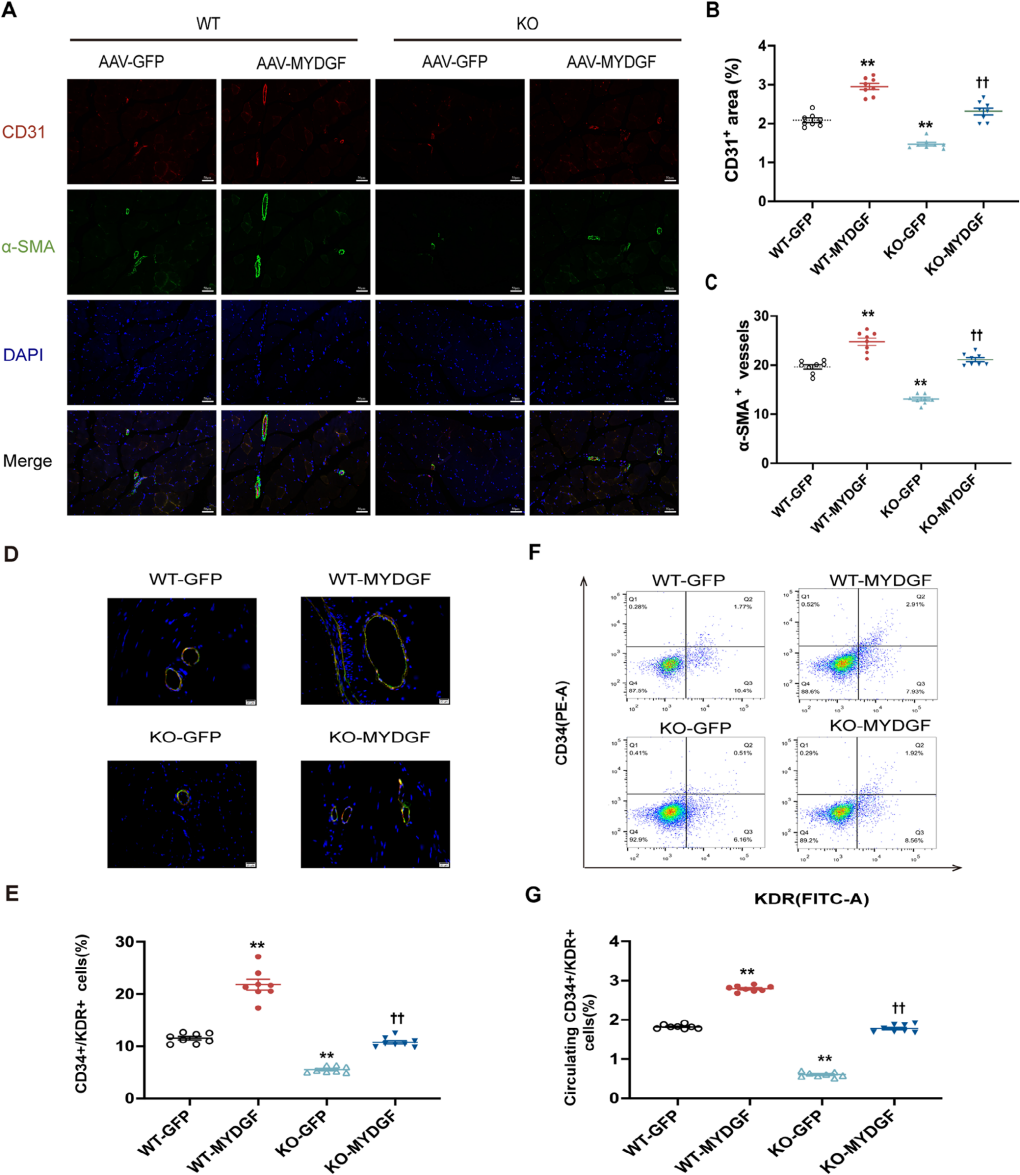
MYDGF enhances angiogenesis in diabetic mice subjected to hindlimb ischemia. (A) Representative images of immunostaining of gastrocnemius muscle sections at 14 days. Scale bar = 50 μm. *n* = 8 per group. (B) Quantification of CD31‐positive area. (C) Quantification of α‐SMA‐positive vessels count. (D) Immunolabelling of muscle sections for KDR (red) and CD34 (green) shows EPCs. Scale bar = 20 μm. *n* = 8 per group. (E) Quantitative analysis of (D). (F) After isolation from blood, the number of circulating EPCs was determined by staining with FITC‐KDR and PE‐CD34 and assessed by flow cytometry (*n* = 8 mice). (G) Quantitative analysis of (F). Data are shown as the mean ± SEM. **p < 0.01 versus WT‐GFP group; ^††^ *p* < 0.01 versus KO‐GFP group.

To elucidate the mechanisms through which MYDGF promotes the restoration of blood perfusion in ischemic lower extremities, immunofluorescence labeling of CD31 and α‐smooth muscle actin (α‐SMA) was performed on ischemic skeletal muscles. Immunofluorescence analysis revealed an obvious decrease in capillary density and arteriolar abundance in MYDGF‐deficient mice compared with their wild‐type counterparts. In contrast, WT‐MYDGF and KO‐MYDGF mice exhibited significantly higher capillary density and arteriolar abundance in the gastrocnemius muscles at 14 days post‐ischemia compared with WT‐GFP and KO‐GFP mice (Figure 2A–C).

Given that EPCs are key regulators of vascularisation, we next investigated whether MYDGF enhances neovascularisation by increasing EPC mobilisation and recruitment. Immunofluorescence analysis revealed a further reduction of CD34^+^/KDR^+^ cells in KO mice relative to WT mice. WT‐MYDGF and KO‐MYDGF mice showed a significant increase in CD34^+^/KDR^+^ cells compared with WT‐GFP and KO‐GFP mice (Figure 2D,E). Consistently, flow cytometry analysis of circulating EPCs demonstrated a higher proportion of CD34^+^/KDR^+^ cells in WT mice compared with MYDGF‐deficient mice and MYDGF restoration further increased their numbers in both WT‐MYDGF and KO‐MYDGF groups (Figure 2F,G). These results suggest that MYDGF promotes EPC mobilisation and homing in DLI mice. Taken together, these findings indicate that MYDGF plays a crucial role in enhancing angiogenesis following ischemic injury in diabetic mice.

### 3.3 MYDGF Enhanced EPC Proliferation and Attenuated Apoptosis In Vitro

Several studies have reported that circulating EPC numbers are reduced in individuals with diabetes [22, 23, 24]. Consistently, our previous work demonstrated decreased EPC counts in diabetic rats [18]. Similarly, our current data found that EPC numbers in circulation and tissues were reduced in diabetic mice, but MYDGF prevented this reduction. To explore whether MYDGF restoration affects EPC proliferation and apoptosis, BM‐EPCs were isolated from WT and KO mice and identified as Dil‐ac‐LDL and lectin double‐positive cells under fluorescence microscopy (Figure S2).

**FIGURE 3:**
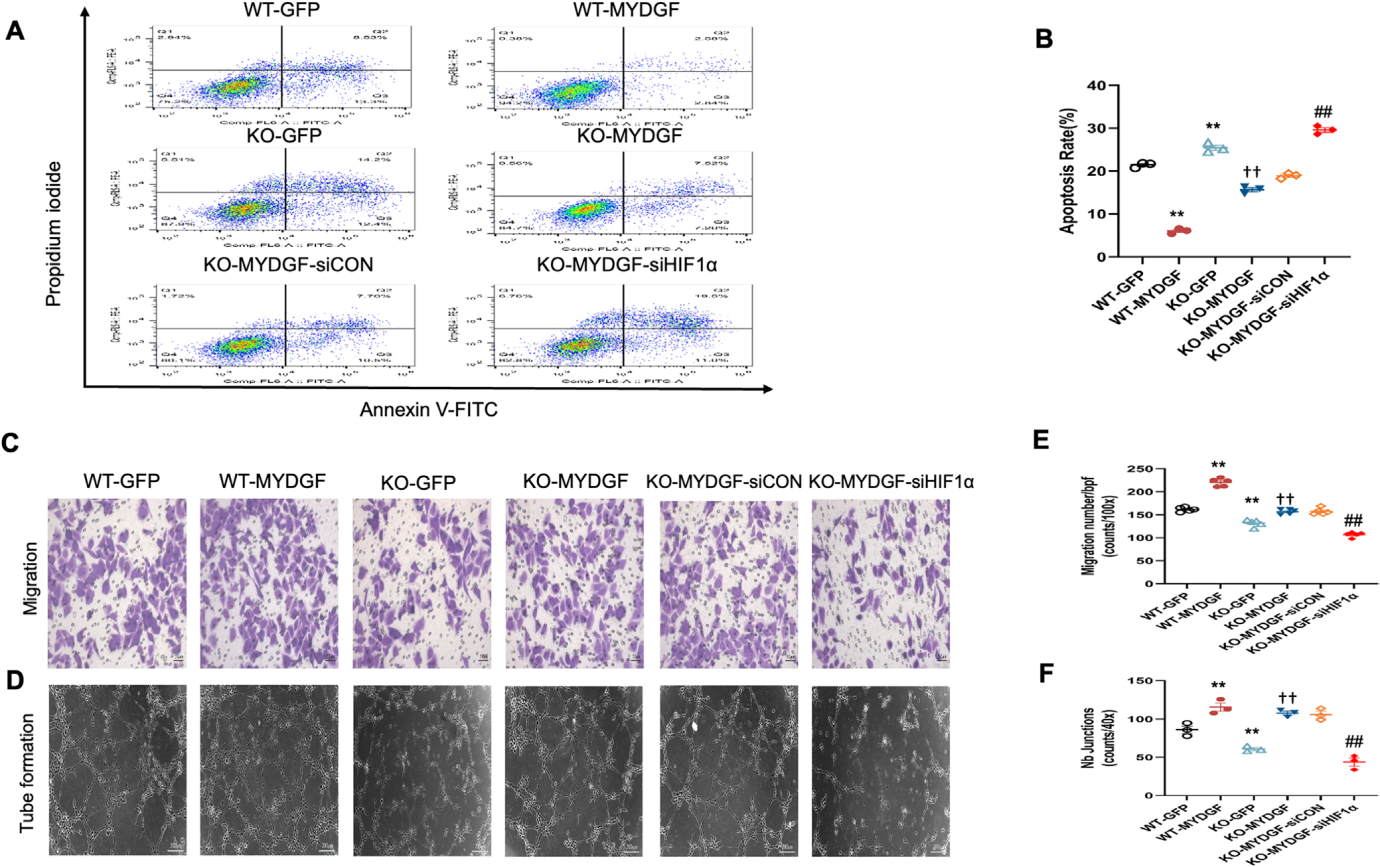
MYDGF improves the angiogenic functions of EPCs in diabetic mice. (A) Representative images of the apoptosis of EPCs from diabetic mice when treated as indicated. Apoptosis rates of EPCs were measured by annexin V–propidium iodide staining and flow cytometry analysis. Apoptotic cells were defined as Annexin V^+^/PI^+^ (Quadrant 2) and Annexin V^+^/PI^−^ (Quadrant 3). *n* = 3 per group. (B) Quantitative analysis of (A). (C) Representative images of migration assay photographs of EPCs from diabetic mice when treated as indicated. Scale bar = 50 μm. *n* = 5 per group. (D) Representative images from the tube formation assay displaying capillary‐like structure formation by EPCs. Scale bar = 200 μm. *n* = 3 per group. (E, F) The associated quantitative analysis of (C, D). Data are presented as mean ± SEM. **p < 0.01 versus WT‐GFP group; ^††^ *p* < 0.01 versus KO‐GFP group; ^##^ *p* < 0.01 versus KO‐MYDGF‐siHIF‐1α group.

**FIGURE 4:**
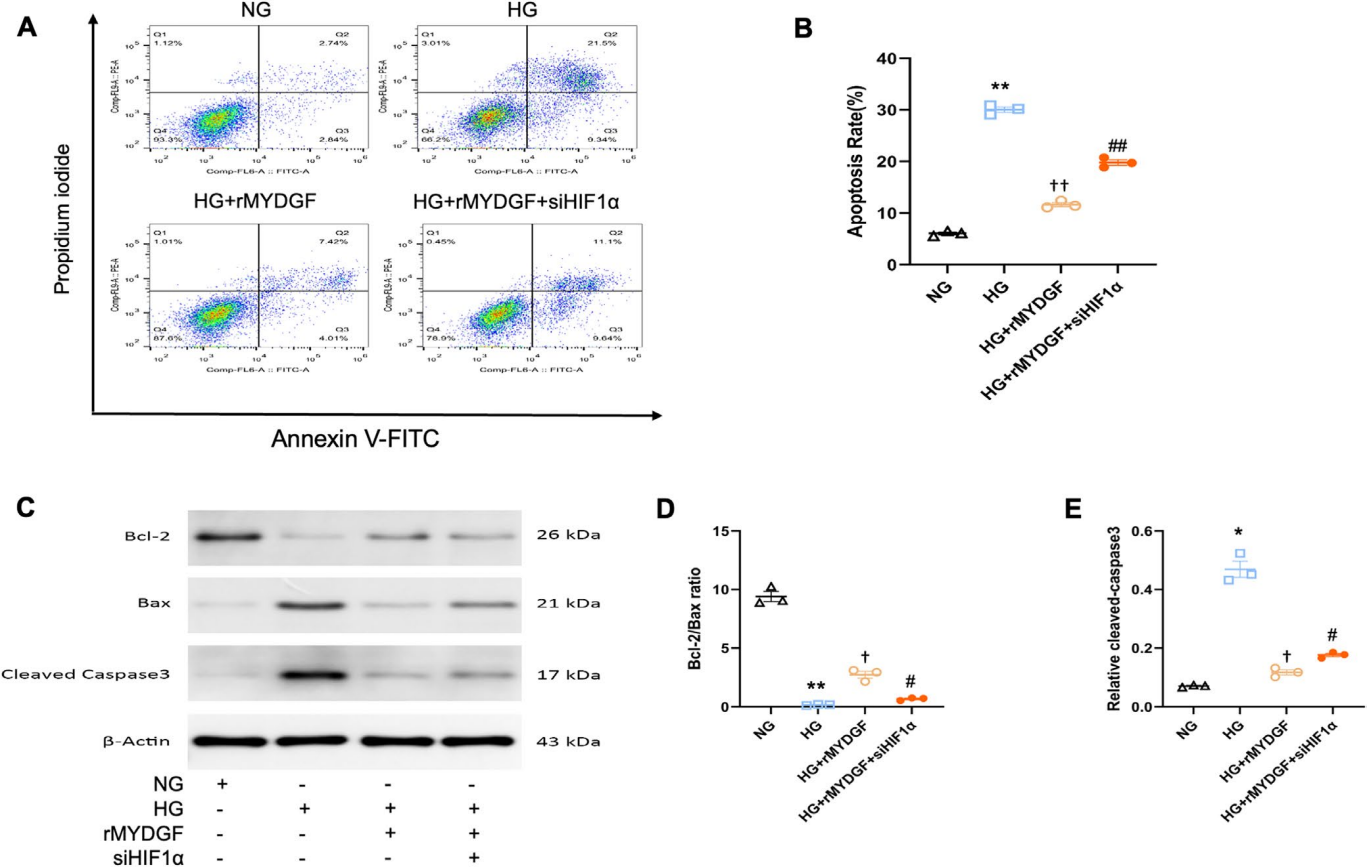
MYDGF alleviates HG‐induced EPC apoptosis in vitro. EPCs were pretreated with or without rMYDGF (100 ng/mL) for 24 h and then treated with or without HG (25 mmol/L) for another 24 h. As indicated, the cells were pretreated with siHIF‐1α for 24 h before rMYDGF was added. In addition, the NG group and HG group without rMYDGF treatment were processed in parallel without changing culture medium. (A) Representative images of apoptotic cells stained with Annexin‐V‐FITC and propidium iodide, which were assessed using flow cytometry. Apoptotic cells were defined as Annexin V^+^/PI^+^ (Quadrant 2) and Annexin V^+^/PI^−^ (Quadrant 3). (B) Quantitative analysis of (A). (C–E) Representative immunoblots and densitometric quantification for the expressions of the proteins Bcl‐2, Bax and cleaved‐caspase3. Data are presented as mean ± SEM of three independent experiments. *p < 0.05, **p < 0.01 versus NG group; ^†^ *p* < 0.05, ^††^ *p* < 0.01 versus HG group; ^#^ *p* < 0.05, ^##^ *p* < 0.01 versus HG+rMYDGF group.

Flow cytometry analysis revealed that the apoptosis rate in KO‐GFP mice was 3.99% higher than in WT‐GFP mice (*p* < 0.01) (Figure 3A,B), indicating that MYDGF deficiency exacerbates BM‐EPC apoptosis. In contrast, AAV‐mediated MYDGF intervention significantly reduced BM‐EPC apoptosis in both WT and KO mice (Figure 3B). To further assess the direct effects of MYDGF on EPC function in vitro, EPCs isolated from non‐diabetic WT mice were treated with rMYDGF. rMYDGF promoted EPC proliferation in a dose‐ and time‐dependent manner (Figure S3), with 100 ng/mL for 48 h selected as the optimal condition. Flow cytometry analysis demonstrated that HG markedly increased the percentage of apoptotic EPCs compared with the NG group, whereas rMYDGF pretreatment significantly attenuated this effect (Figure 4A,B). Western blotting further confirmed that rMYDGF treatment increased expression of the anti‐apoptotic protein Bcl‐2 and decreased expression of pro‐apoptotic proteins Bax and cleaved caspase‐3 (Figure 4C–E). Overall, these results suggest that MYDGF protects EPCs from HG‐induced apoptosis and promotes their proliferation, supporting its critical role in maintaining EPC function under diabetic conditions.

### 3.4 MYDGF Restoration Enhanced BM‐EPC Angiogenic Functions In Vitro

Our in vivo data demonstrated that MYDGF deficiency impaired vessel density and EPC mobilisation, whereas MYDGF restoration markedly improved angiogenesis and perfusion in DLI. We therefore investigated whether these beneficial effects were mediated through modulation of EPC angiogenic activity.

TEM, a key step in EPC homing to sites of vascular repair, was assessed using a transwell migration assay and angiogenic capacity was evaluated using a Matrigel tube formation assay. These analyses revealed that BM‐EPCs isolated from KO‐GFP mice exhibited significantly reduced migratory capacity and impaired tube‐like structure formation compared with those from WT‐GFP mice, indicating that MYDGF deficiency compromises EPC angiogenic function. In contrast, AAV‐mediated MYDGF restoration significantly enhanced both migration and tube formation in BM‐EPCs derived from WT and KO mice (Figure 3C–F).

**FIGURE 5:**
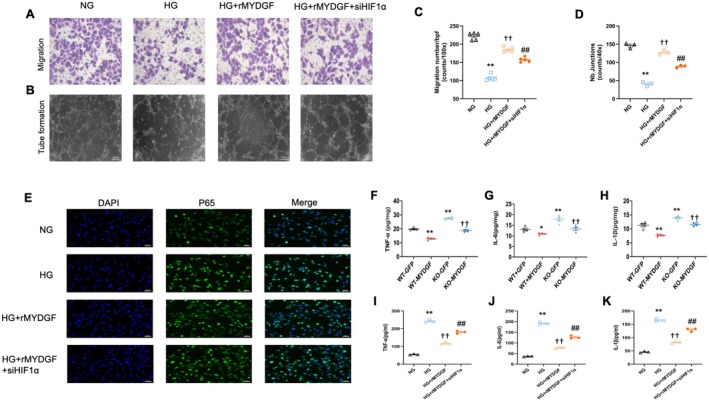
MYDGF improves EPC tube formation and migration in vitro and reduces the levels of inflammatory cytokines in vivo and in vitro. The pre‐treatment of EPCs is the same as above. (A) Representative images from the Transwell assay showing EPCs migration. Five independent experiments were performed. Scale bar = 50 μm. (B) Representative images from the tube formation assay displaying capillary‐like structure formation by EPCs. Three independent experiments were performed. Scale bar = 200 μm. (C, D) The corresponding quantitative analysis of (A, B). (E) The nuclear translocation of NF‐κB p65 in the EPCs was assayed using an immunofluorescence assay. Four independent experiments were performed. Scale bar = 50 μm. (F–H) The expression levels of pro‐inflammatory factors (TNF‐α, IL‐6 and IL‐1β) in gastrocnemius muscle tissues using ELISA assay. *n* = 4 per group. (I–K) Effects of co‐incubation of EPCs with rMYDGF (100 ng/mL) or rMYDGF (100 ng/mL) together with siHIF‐1α on HG‐induced production of TNF‐α, IL‐6 and IL‐1β. Three independent experiments were performed. Data are presented as mean ± SEM. **p < 0.01 versus NG group; ^††^ *p* < 0.01 versus HG group; ^##^ *p* < 0.01 versus HG+rMYDGF group. *p < 0.05, **p < 0.01 versus WT‐GFP group; ^††^ *p* < 0.01 versus KO‐GFP group.

Angiogenic function was further evaluated in vitro using EPCs isolated from non‐diabetic WT mice. As shown in Figure 5A–D, exposure to HG markedly suppressed EPC migration and tube formation compared with NG conditions. Pretreatment with rMYDGF significantly rescued these defects, restoring both migratory and tube‐forming capacities under HG conditions. Taken together, these findings indicate that MYDGF promotes EPC angiogenic function and may represent a therapeutic strategy for enhancing angiogenesis in hyperglycemic environments.

### 3.5 MYDGF Attenuated Inflammatory Responses In Vivo and In Vitro

Inflammation is a critical contributor to ischemic tissue injury, particularly during the early stages of disease progression [25]. To determine whether MYDGF modulates inflammatory responses in DLI, ELISA analyses were performed on gastrocnemius muscle tissues from the DLI mouse model. KO‐GFP mice showed significantly elevated levels of TNF‐α, IL‐6 and IL‐1β compared with WT‐GFP mice. However, AAV‐MYDGF treatment significantly reduced the expression of these pro‐inflammatory cytokines in both WT and KO mice (*p* < 0.01) (Figure 5F–H). Consistent with the in vivo findings, in vitro experiments demonstrated that rMYDGF pretreatment markedly inhibited HG‐induced nuclear translocation of nuclear factor‐kappa B (NF‐κB) p65 in EPCs (Figure 5E). Furthermore, EPCs exposed to HG secreted significantly higher levels of TNF‐α, IL‐6 and IL‐1β than those cultured under NG conditions, whereas rMYDGF treatment significantly suppressed HG‐induced cytokine production (Figure 5I–K). Together, these results demonstrate that MYDGF enhances EPC angiogenic capacity while simultaneously attenuating inflammatory responses, thereby contributing to improved vascular repair under diabetic ischemic conditions.

### 3.6 AKT/HIF‐1α Signalling Was Required for the Protective Effects of MYDGF in DLI

**FIGURE 6:**
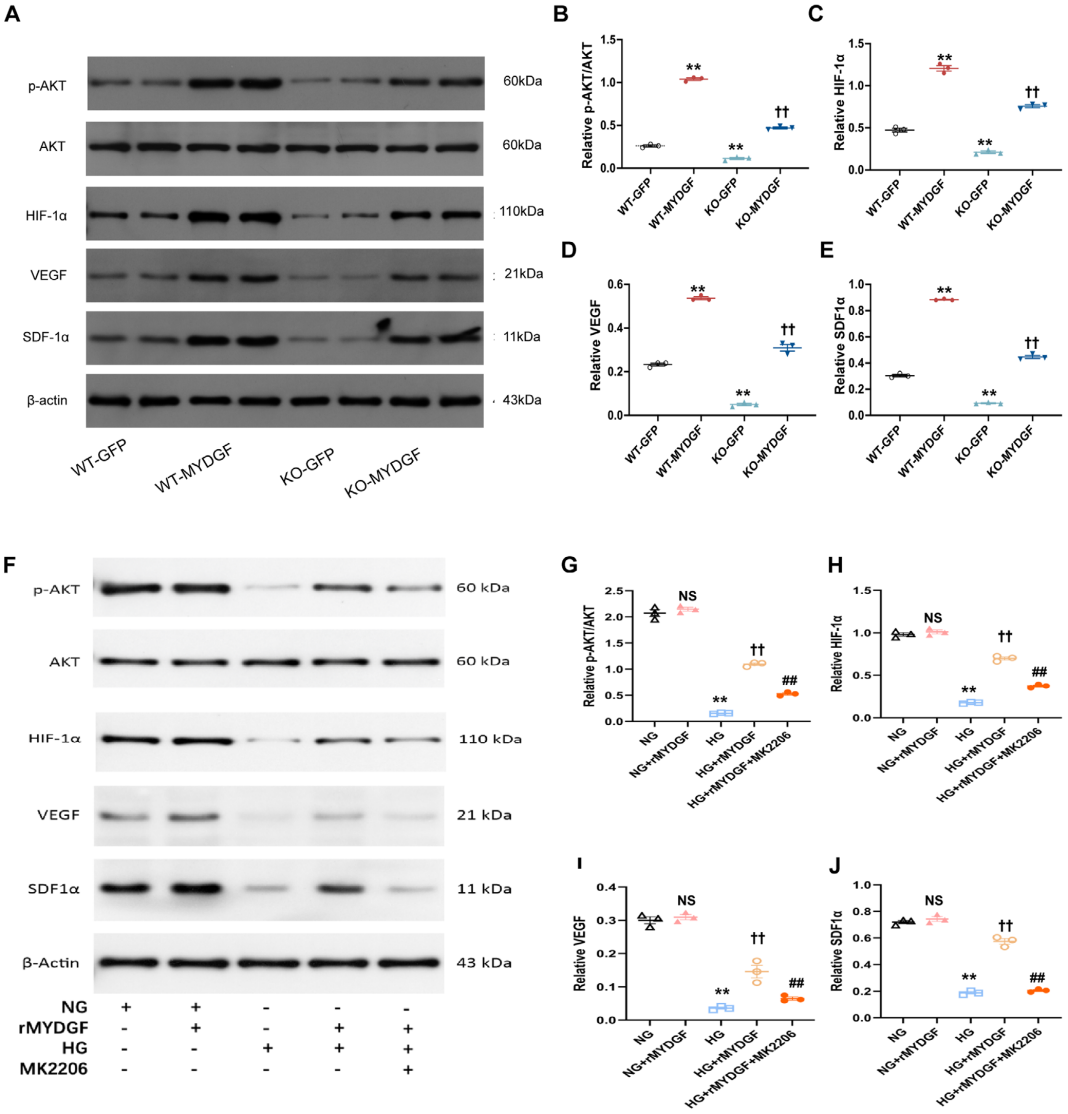
MYDGF activates AKT/HIF‐1α pathway in vivo and in vitro (A) Western blot analysis of p‐AKT, AKT, HIF‐1α, VEGF and SDF‐1α normalised to β‐Actin protein in the ischemic muscle from mice in various treatment groups. (B–E) Quantitative analysis of (A). **p < 0.01 versus WT‐GFP group; ^††^ *p* < 0.01 versus KO‐GFP group. (F) Representative immunoblots and densitometric quantification for expressions of p‐AKT, HIF‐1α, VEGF and SDF‐1α in EPC cells exposure to rMYDGF (100 ng/mL) for 1 h with or without AKT inhibitor MK2206 (10 μM before rMYDGF for 1 h). (G–J) Quantitative analysis of (F). Data are presented as mean ± SEM of 3 independent experiments. NS (not significant) versus NG group; **p < 0.01 versus NG group; ^††^ *p* < 0.01 versus HG group; ^##^ *p* < 0.01 versus HG+rMYDGF group.

As described above, MYDGF restoration enhanced vascularisation and promoted functional recovery in DLI. We next explored the signalling pathways underlying these beneficial effects. Angiogenesis is regulated by multiple signalling cascades activated by diverse angiogenic factors. Among these, HIF‐1α is a central regulator of ischemia‐induced angiogenesis, controlling the transcription of downstream targets such as VEGF and SDF‐1α [26, 27]. A previous study has shown that MYDGF deficiency impairs wound healing through disruption of HIF‐1α signalling [13]. Therefore, we examined whether MYDGF exerts its effects via this pathway in DLI. Western blot analysis revealed significantly reduced HIF‐1α protein levels in ischemic muscle tissues from MYDGF‐deficient mice relative to WT mice. Conversely, HIF‐1α expression was markedly increased in both WT‐MYDGF and KO‐MYDGF mice relative to their respective AAV‐GFP–treated counterparts. Consistent with HIF‐1α activation, MYDGF restoration significantly upregulated VEGF and SDF‐1α expression in ischemic muscle tissues (Figure 6A–E).

Because HIF‐1α activity is regulated by multiple upstream kinases, we examined the phosphorylation status of key signalling molecules, including AKT, AMPK and ERK1/2. MYDGF treatment selectively enhanced AKT phosphorylation in ischemic tissues (Figure 6A–E), whereas phosphorylation of ERK1/2 and AMPK remained unchanged (Figure S4), indicating pathway‐specific activation of AKT signalling.

These findings were further validated in cultured EPCs. HG conditions markedly reduced AKT phosphorylation, HIF‐1α protein levels and the expression of VEGF and SDF‐1α. Pretreatment with rMYDGF partially rescued these defects. Importantly, co‐incubation with the AKT inhibitor MK2206 abolished rMYDGF‐induced AKT activation and suppressed the upregulation of HIF‐1α, VEGF and SDF‐1α (Figure 6F–J).

**FIGURE 7:**
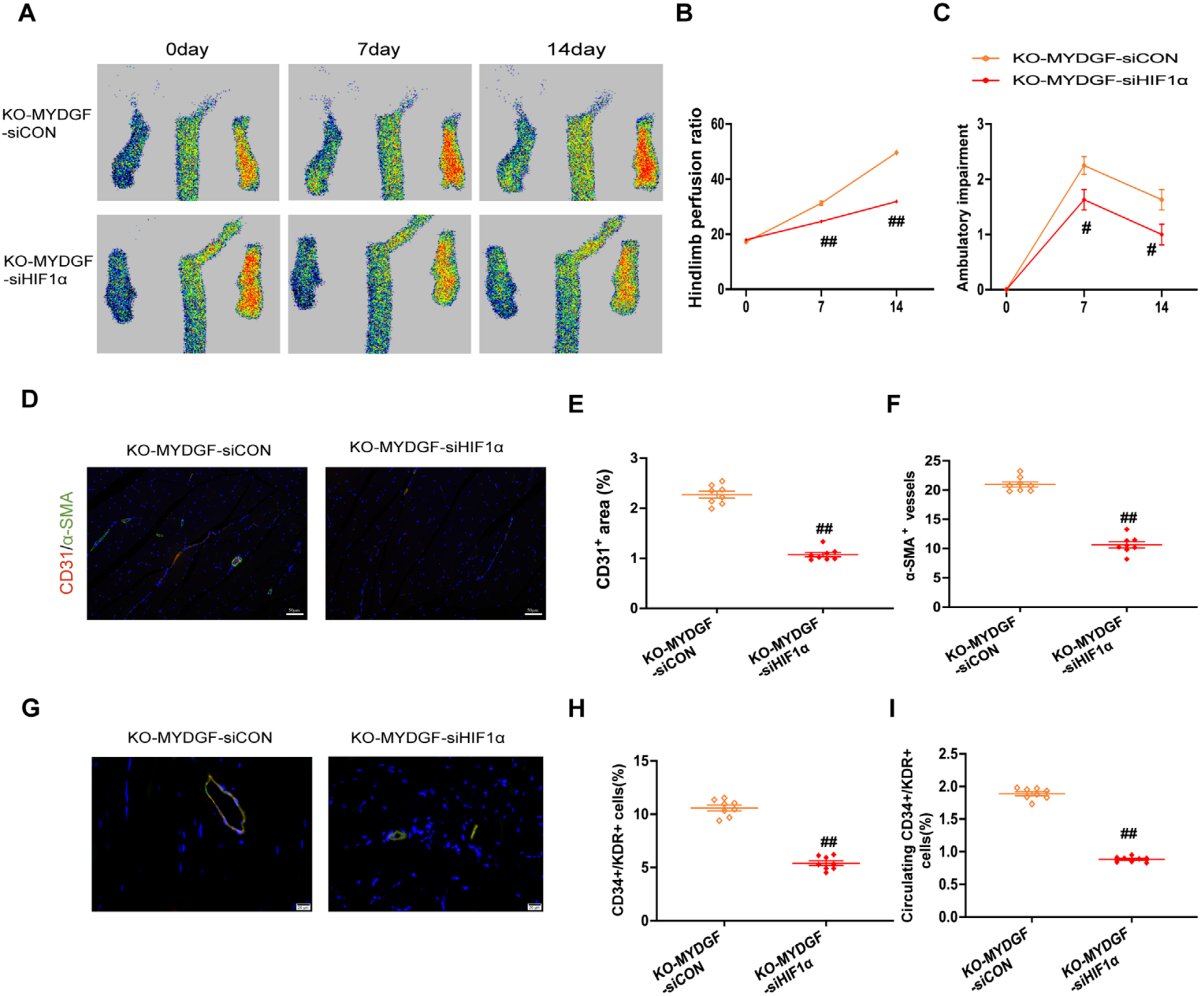
siHIF‐1α abrogates the positive effects of MYDGF on angiogenesis and impairs blood flow in vivo. KO‐MYDGF mice were further randomised to siCon and siHIF‐1α groups (*n* = 8 in each group). (A) Representative LDPI images display the time course of ischemic limb perfusion. (B) Quantitative analysis of (A). (C) Ambulatory impairment was scored at each time point. (D) Representative images of immunostaining of gastrocnemius muscle sections at 14 days. Scale bar = 50 μm. *n* = 8 per group. (E) Quantification of CD31‐positive area. (F) Quantification of α‐SMA‐positive vessels count. (G) Immunolabelling of muscle sections for KDR (red) and CD34 (green) shows EPCs in KO‐MYDGF‐siCON and KO‐MYDGF‐siHIF‐1α groups. (H) Quantitative analysis of (G). (I) The number of circulating EPCs in KO‐MYDGF‐siCON and KO‐MYDGF‐siHIF‐1α groups were assessed by flow cytometry. Data are presented as mean ± SEM. ^#^ *p* < 0.05, ^##^ *p* < 0.01 versus KO‐MYDGF‐siCON group.

To further define the role of HIF‐1α in MYDGF‐mediated vascular repair, we performed RNA interference‐mediated HIF‐1α silencing in vivo and in vitro. First, we evaluated the silencing efficiency; as expected, we observed significant decreases in HIF‐1α protein levels compared with the siCon group both in vivo and in vitro (Figure S5). LDPI demonstrated that HIF‐1α knockdown significantly impaired post‐ischemic blood flow recovery in diabetic hindlimbs, despite MYDGF restoration (Figure 7A–C). Immunofluorescence analyses revealed that suppression of HIF‐1α reduced capillary density and arteriolar number in ischemic muscles (Figure 7D–F). Consistently, the KO‐MYDGF+siHIF‐1α group showed reduced EPC mobilisation and homing to ischemic tissues (Figure 7G–I), indicating impaired ischemia‐induced EPC recruitment. At the cellular level, HIF‐1α silencing increased apoptosis of BM‐EPCs and reduced their migratory and tube‐forming capacities (Figure 3A–F). In cultured EPCs, siHIF‐1α partially abolished the protective effects of MYDGF on apoptosis (Figure 4), migration and tube formation (Figure 5A–D) and inflammatory responses (Figure 5E,I–K). Taken together, these results demonstrated that the anti‐apoptotic, pro‐angiogenic and anti‐inflammatory effects of MYDGF are likely mediated via activation of the AKT/HIF‐1α signalling pathway.

## 4 Discussion

In this study, we investigated the role of MYDGF in DLI and impaired angiogenesis following vascular injury. The major findings were as follows: (1) MYDGF enhanced vascularisation and accelerated blood flow recovery in DLI; (2) MYDGF promoted EPC angiogenic functions, including tube formation and migration and protected EPCs from apoptosis; (3) MYDGF suppressed excessive inflammatory responses in vivo and in vitro; and (4) these beneficial effects were mediated, at least in part, by the activation of the AKT/HIF‐1α signalling pathway. To our knowledge, this is the first demonstration that MYDGF restoration rescues EPCs from diabetes‐induced damage and significantly improves vascularisation in DLI.

Vascular complications in diabetes are characterised by impaired vascular remodelling, decreased responsiveness to ischemic or hypoxic stimuli, defective neovascularisation and insufficient endothelial regeneration [28]. These abnormalities underscore the urgent need for therapeutic strategies that restore angiogenic capacity and enhance blood flow in diabetic patients. Our findings demonstrated that MYDGF treatment markedly increased vascular density in ischemic tissues of diabetic mice and substantially improved perfusion in the affected limb. Conversely, MYDGF deficiency exacerbated impairments in capillary density, arteriolar abundance and DLI recovery compared with diabetic WT mice. Both loss‐ and gain‐of‐function approaches suggest that MYDGF plays a critical role in mitigating DLI. Collectively, these results establish MYDGF as a key regulator of revascularisation and functional recovery following ischemic injury in diabetes.

Patients with type 1 or type 2 diabetes exhibit reduced numbers and impaired angiogenic function of EPCs, which contribute causally to the development and progression of nearly all diabetic complications [29]. Our previous studies demonstrated that MYDGF preserved endothelial function and reduced endothelial apoptosis in atherosclerotic mice [12]. In the present in vivo and in vitro experiments, MYDGF deficiency markedly exacerbated the reduction in EPC homing to ischemic sites, impaired BM‐EPC migratory and tube‐forming capacity and increased BM‐EPC apoptosis. In contrast, MYDGF overexpression significantly enhanced EPC mobilisation and recruitment, improved BM‐EPC migration and tube formation and attenuated apoptosis in both diabetic WT and KO mice. Consistently, in vitro exposure of EPCs to HG induced elevated apoptotic protein expression and increased apoptosis rates, which were significantly ameliorated by rMYDGF treatment. These results highlight the critical role of MYDGF in maintaining EPC function under diabetic conditions.

Limb ischemia and ischemia–reperfusion are increasingly recognised to trigger inflammatory cytokine cascades [30]. These cytokines contribute to enhanced platelet aggregation and intimal thickening, exacerbating arterial narrowing and thrombus formation and further compromising blood supply to the lower limbs [31, 32]. In this study, myeloid cell‐derived MYDGF mitigated inflammatory responses in gastrocnemius muscle tissue from DLI mice. Similarly, rMYDGF treatment attenuated HG‐induced NF‐κB p65 nuclear translocation and suppressed pro‐inflammatory cytokine production in EPCs. Collectively, these findings indicate that the anti‐inflammatory effects of MYDGF substantially contribute to its protective role in DLI.

Recent studies have highlighted the pivotal role of the HIF‐1α pathway in regulating vessel density and perfusion in DLI [18, 33, 34]. Furthermore, MYDGF has been shown to interact with HIF‐1α signalling to modulate neutrophil accumulation in response to tissue injury [13]. In the present study, we observed that HIF‐1α levels were significantly reduced in both DLI mice and HG‐treated EPCs, whereas MYDGF replenishment restored its expression. HIF‐1α activation is regulated by multiple upstream kinases, and our results demonstrated that MYDGF exerted its biological activity primarily via the AKT/HIF‐1α pathway. Moreover, MYDGF upregulated the expression of downstream angiogenic factors, including VEGF and SDF‐1α. Importantly, MYDGF‐induced angiogenesis was markedly impaired in siHIF‐1α mice and HIF‐1α silencing partially abrogated the protective effects of MYDGF on EPC survival and function in vitro, indicating that HIF‐1α downregulation attenuates the pro‐angiogenic effect of MYDGF. Collectively, these findings establish that activation of the AKT/HIF‐1α cascade is essential for the beneficial effects of MYDGF.

Several limitations should be mentioned here. First, although we demonstrate that MYDGF promotes angiogenesis through activation of the canonical AKT/HIF‐1α signalling axis, the upstream receptor(s) mediating this effect remain unidentified. Additionally, it remains unclear whether MYDGF acts at the transcriptional or protein level, or both, to regulate the expression and activity of key components within this pathway. Furthermore, in the present study, we identified murine EPCs as CD34^+^/KDR^+^ cells, a marker combination adopted in multiple recent studies. However, alternative markers such as Sca‐1 and CD133 have been used in various combinations and marker selection may significantly affect the identified cell population and its functional properties. Thus, further studies are required to address these gaps.

In summary, we identified a previously unrecognised role for the paracrine factor MYDGF in vascular repair. MYDGF promoted angiogenesis and restored blood flow in DLI by increasing EPC number and enhancing EPC function via AKT/HIF‐1α signalling. These findings provide new mechanistic insights and suggest the translational potential of MYDGF as a therapeutic strategy for improving vascularisation and perfusion in patients with diabetes.

## Author Contributions

**Biying Meng:** methodology, investigation. **Jingwen Ye:** investigation, data curation. **Bilin Zhang:** data curation, formal analysis. **Wen Mei:** writing – original draft, methodology, investigation. **Haizhao Luo:** data curation, investigation. **Yijiang Hou:** methodology, data curation. **Biao Zhu:** data curation, formal analysis. **Yi Shu:** data curation, formal analysis. **Jiajia Zhang:** writing – review and editing, funding acquisition, project administration.

## Funding

This work was supported by Natural Science Foundation of Hubei Province of China (Grant 2024AFB1072), the Medical Science and Technology Research Foundation of Guangdong Province (Grant A2023325) and National Natural Science Foundation of China (Grant NSFC 82300931).

## Ethics Statement

Animal experiments involved were approved by the Animal Ethics Committee of the General Hospital of Central Theatre Command, Wuhan, China. (Animal Ethics Approval No: 2023001) and were conducted under the Guidelines for Animal Experimentation. The date of the ethics approval was on March 10, 2023.

## Conflicts of Interest

The authors declare no conflicts of interest.

## Supporting information


**Figure S1:** MYDGF is suppressed under diabetic conditions. (A) Experimental scheme of establishment of T2DM in mice and femoral artery ligation to mice. (B) the plasma MYDGF concentration was detected by LC–MS (*n* = 8 mice). **p < 0.01 versus Non‐DM group. (C) Representative images illustrating blood flow in hindlimb paws, as monitored at 0, 7 and 14 days. (D) Quantification of blood flow recovery at 0, 7 and 14 days, assessed by the ischemic to non‐ischemic limb perfusion ratio. **p < 0.01 versus Non‐DM group. (E) Plasma MYDGF levels were determined by LC–MS (*n* = 8 mice per group). Data are shown as the mean ± SEM. **p < 0.01 versus WT‐GFP group; ^††^ *p* < 0.01 versus KO‐GFP group.
**Figure S2:** Characterisation of BM‐EPCs.
**Figure S3:** The different conditions regulate EPCs proliferation in vitro.
**Figure S4:** MYDGF activates AKT/HIF1α pathway in vivo.
**Figure S5:** The HIF‐1α expression after silencing by siRNA in vivo and in vitro.

## Data Availability

The data that support the findings of this study are available from the corresponding author upon reasonable request.
