# Supplementary material for: Myeloid‐Derived Growth Factor Improves Endothelial Progenitor Cell Function and Angiogenesis in Mice With Diabetic Hindlimb Ischemia by Activating Akt/HIF‐1α Signalling Pathway
Source: J Cell Mol Med. 2026 Jul 14;30(13):e71287. doi: 10.1111/jcmm.71287 (PMC13367132; doi:10.1111/jcmm.71287)
Supplement: Supplementary file 1 — Figure S1: MYDGF is suppressed under diabetic conditions. (A) Experimental scheme of establishment of T2DM in mice and femoral artery ligation to mice. (B) the plasma MYDGF concentration was detected by LC–MS (n = 8 mice). **p < 0.01 versus Non‐DM group. (C) Representative images illustrating blood flow in hindlimb paws, as monitored at 0, 7 and 14 days. (D) Quantification of blood flow recovery at 0, 7 and 14 days, assessed by the ischemic to non‐ischemic limb perfusion ratio. **p < 0.01 versus Non‐DM group. (E) Plasma MYDGF levels were determined by LC–MS (n = 8 mice per group). Data are shown as the mean ± SEM. **p < 0.01 versus WT‐GFP group; †† p < 0.01 versus KO‐GFP group. Figure S2: Characterisation of BM‐EPCs. Figure S3: The different conditions regulate EPCs proliferation in vitro. Figure S4: MYDGF activates AKT/HIF1α pathway in vivo. Figure S5: The HIF‐1α expression after silencing by siRNA in vivo and in vitro. [file JCMM-30-e71287-s001.docx]

**SUPPLEMENTAL MATERIAL**

**Supplemental table 1. Sequences of siRNAs**

| Target Gene | siRNA sequence (5’−3’) |
| --- | --- |
| HIF-1α-1 | AAGGAUGCAAAUCUAGUGAAC |
| HIF-1α-2 | AAGCACAGUUACAGUAUUCCA |
| HIF-1α-3 | AAGUUCACCUGAGCCUAAUAG |
| HIF-1α-NC | AAGUCUAGAUGAAAUGGCAAC |

**
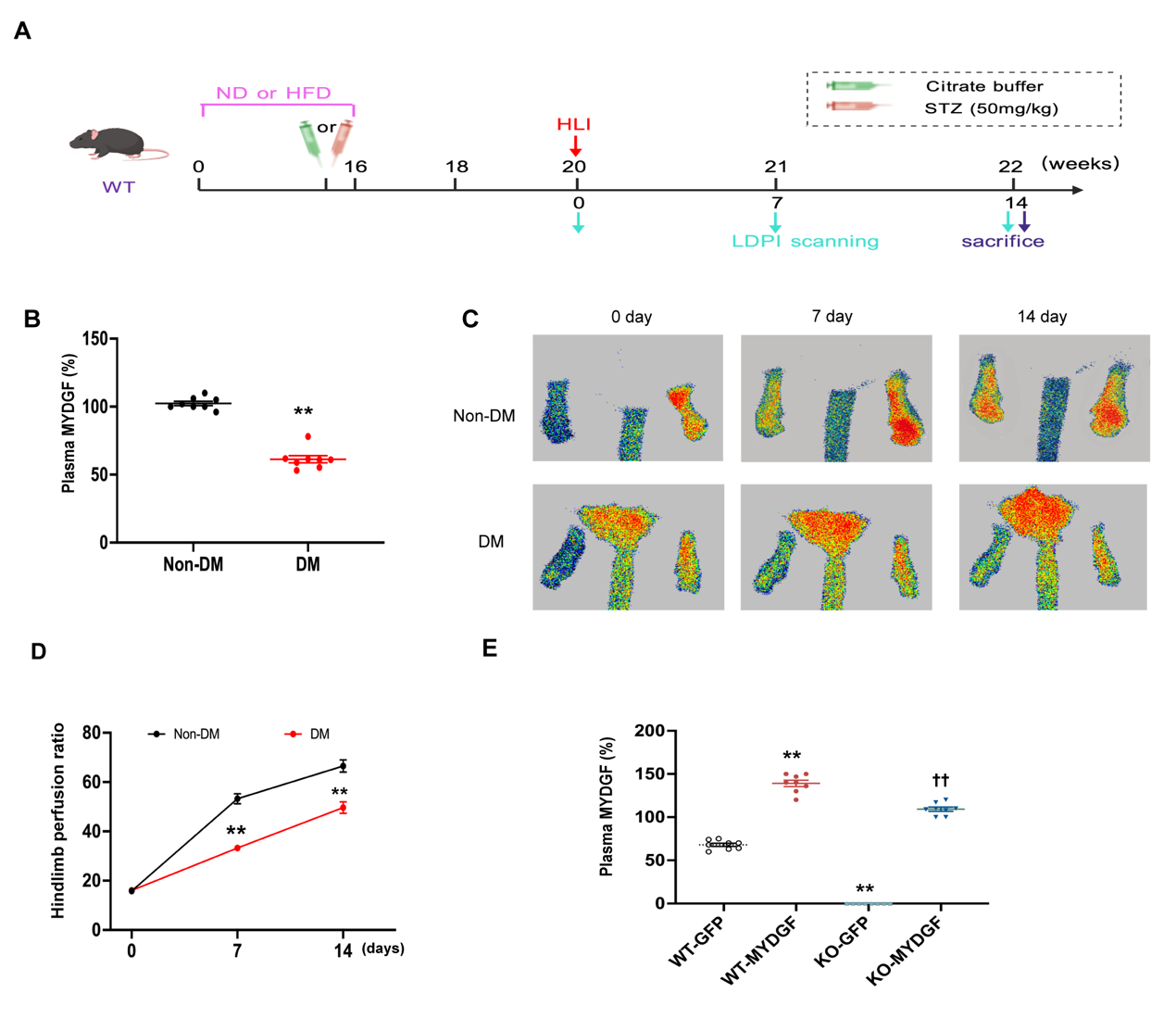
**

**Supplementary figure 1. MYDGF is suppressed under diabetic conditions.** (A) Experimental scheme of establishment of T2DM in mice and femoral artery ligation to mice. (B) the plasma MYDGF concentration was detected by LC-MS (n = 8 mice). ***p* < 0.01 vs Non-DM group. (C) Representative images illustrating blood flow in hindlimb paws, as monitored at 0, 7, and 14 days. (D) Quantification of blood flow recovery at 0, 7, and 14 days, assessed by the ischemic to non-ischemic limb perfusion ratio. ***p* < 0.01 vs Non-DM group. (E) Plasma MYDGF levels were determined by LC-MS (n=8 mice per group). Data are shown as the mean ± SEM. ***p*<0.01 vs. WT-GFP group; ^††^*p*< 0.01 vs. KO-GFP group.

**
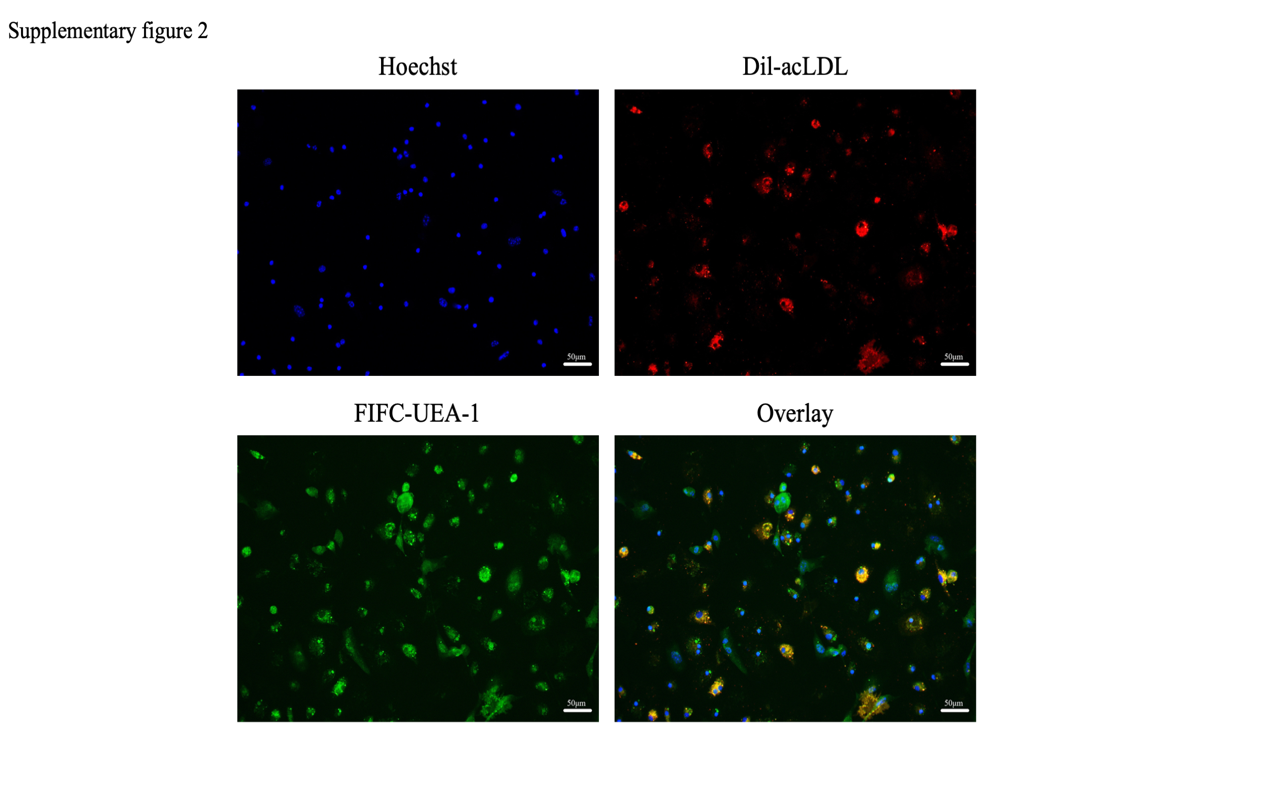
**

**Supplementary figure 2. Characterization of BM-EPCs.**

BM-EPCs were identified as Dil-acLDL (red) and lectin (green) double-positive cells under the fluorescence microscope. Nuclei were stained with Hoechst (blue). Scale bar = 50 μm.


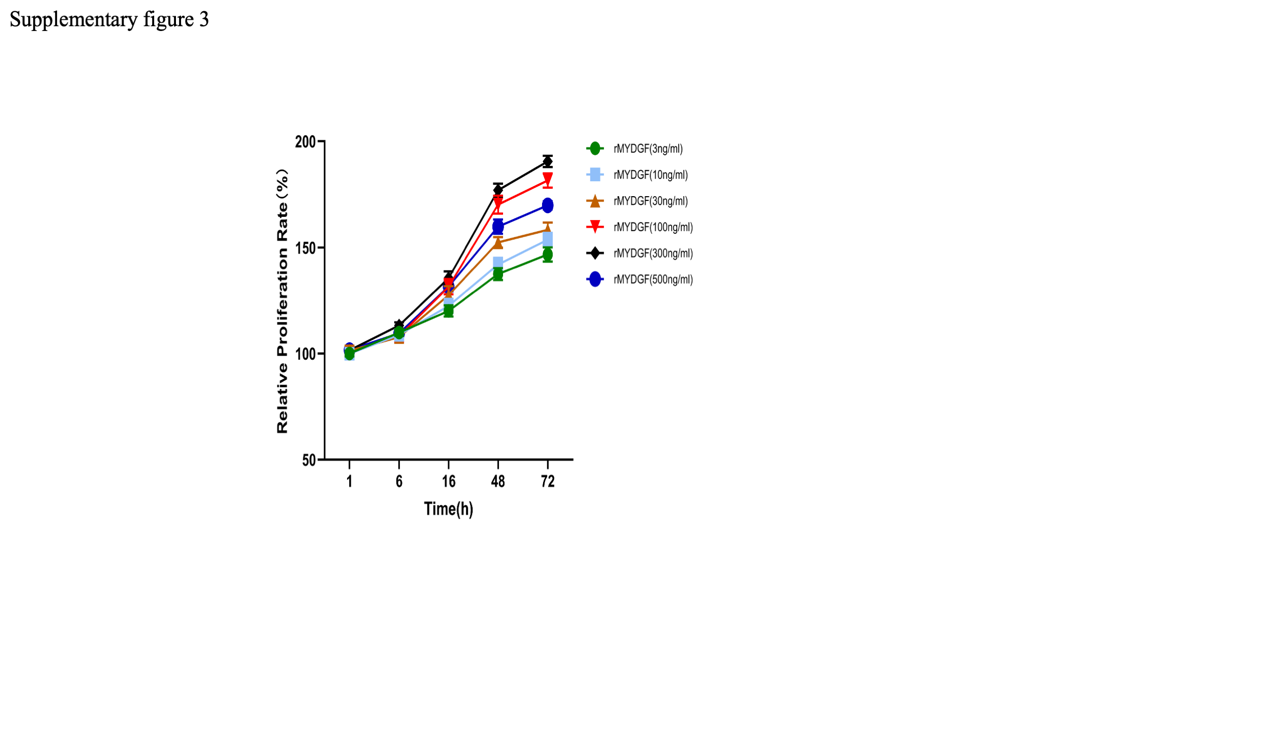


**Supplementary figure 3. The different conditions regulate EPCs proliferation in vitro.**

EPCs were pretreated by rMYDGF with different time and dose. The cell relative proliferation rate was determined by Cell Counting Kit-8 (CCK8).


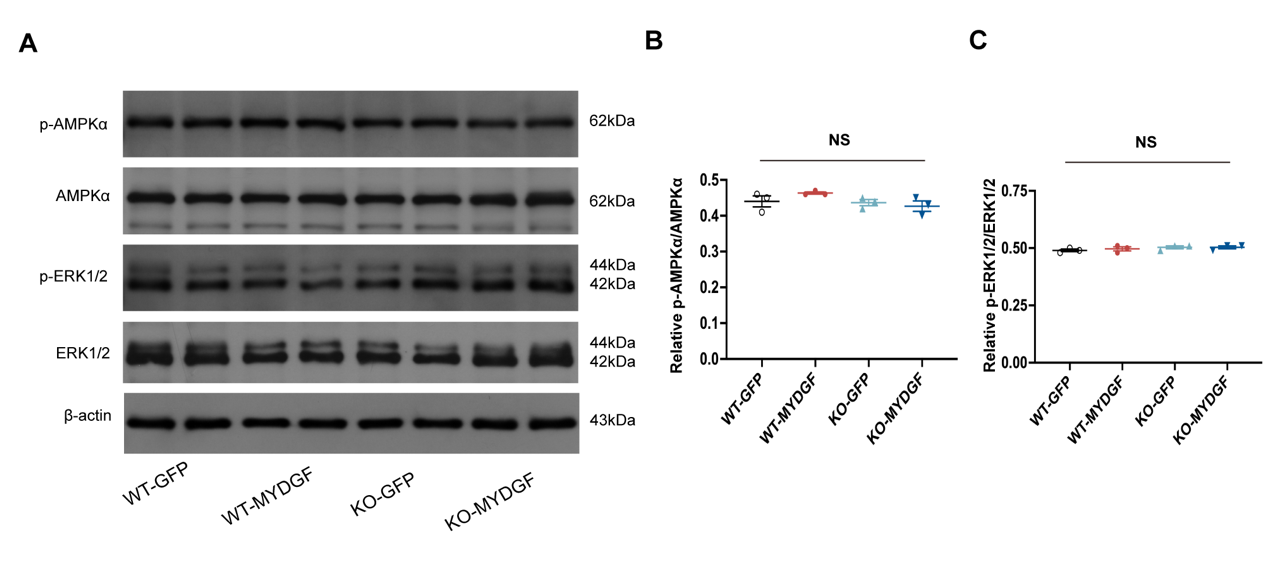


**Supplementary figure 4.** **MYDGF activates AKT/HIF1α** **pathway *in vivo***

(A) Western blot analyses for p-AMPKα, AMPKα, p-ERK1/2 and ERK1/2 in different groups. Supplementary figure 4A and Figure 6A share the same β-actin control, as they originate from the same experimental batch and the same original membrane. (B-C) Bar graphs show averages of the ratios (phosphorylated proteins to total proteins) of the band intensities (n = 3 per group). NS: no significance.

**
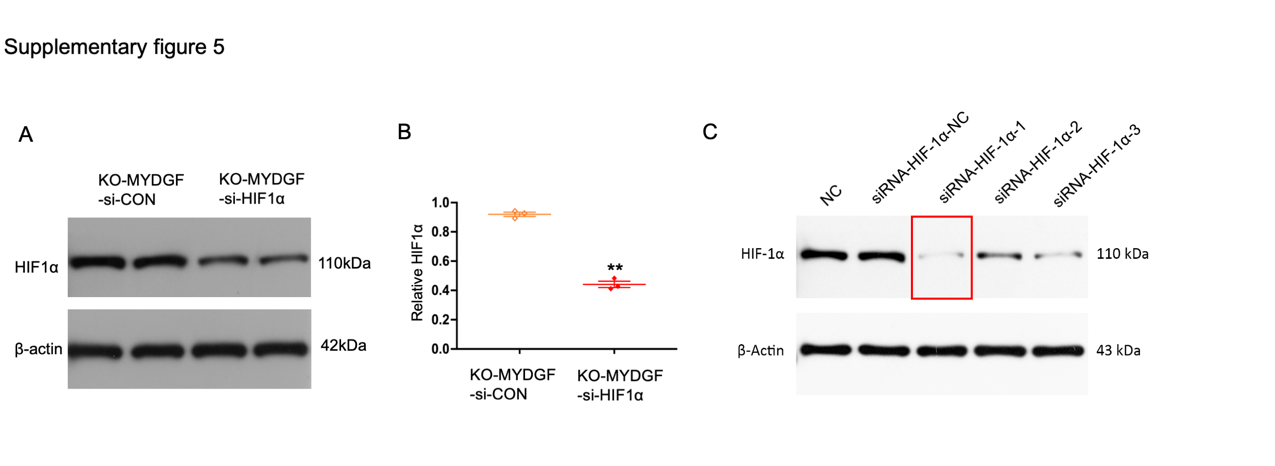
**

**Supplementary figure 5. The HIF-1α expression after silencing by siRNA *in vivo* and *in vitro***

(A) The protein expression of HIF-1α in different groups that treated with siCon or siHIF-1α (n = 3 per group). (B) Quantitative analysis of (A). (C) EPCs were infected with siRNA-HIF-1α-1, siRNA-HIF-1α-2 or siRNA-HIF-1α-3 for 24h, and NC and siRNA-HIF-1α-NC were used as control group. the efficiency of HIF-1α knockdown was determined by Western blot. We ultimately chose siRNA-HIF-1α-1. NC, normal control. Data are presented as mean ± SEM. ** *p*<0.01 vs. siCon group.
